# Structural, Morphological and Electrochemical Characterization of Hydrothermally Fabricated PdNiCo and PdNiCo-rGO Alloys for Use as Counter Electrode Catalysts in DSSC

**DOI:** 10.3390/ma12193256

**Published:** 2019-10-06

**Authors:** Edson Meyer, Johannes Mbese, Dorcas Mutukwa, Nyengerai Zingwe

**Affiliations:** 1 Fort Hare Institute of Technology, University of Fort Hare, Alice 5700, South Africa; emeyer@ufh.ac.za (E.M.); dmutukwa@ufh.ac.za (D.M.); 2 Department of Chemistry, University of Fort Hare, Alice 5700, South Africa; jmbese@ufh.ac.za

**Keywords:** palladium, alloy, counter electrodes, dye sensitized solar cell, catalyst

## Abstract

The hydrothermal fabrication and characterization of ternary palladium alloys PdNiCo and PdNiCo-rGO, which could be potential replacements to the expensive and corrosion susceptible platinum counter electrode in dye sensitized solar cells is hereby reported in this article. The synergy created by combining three metallic elements as well as the effect of carbon supports was investigated. The as-synthesized alloys consisted of agglomerated spherical particles. Comparison of the electrochemical analysis data showed that PdNiCo-rGO counter electrode could be a potential replacement for the platinum counter electrode with reduction current density, peak to peak potential difference, charge transfer resistance and power conversion efficiency of 21 mA∙cm^−2^, 0.12 mV, 0.726 Ω and 4.36% respectively.

## 1. Introduction

**Figure 1:**
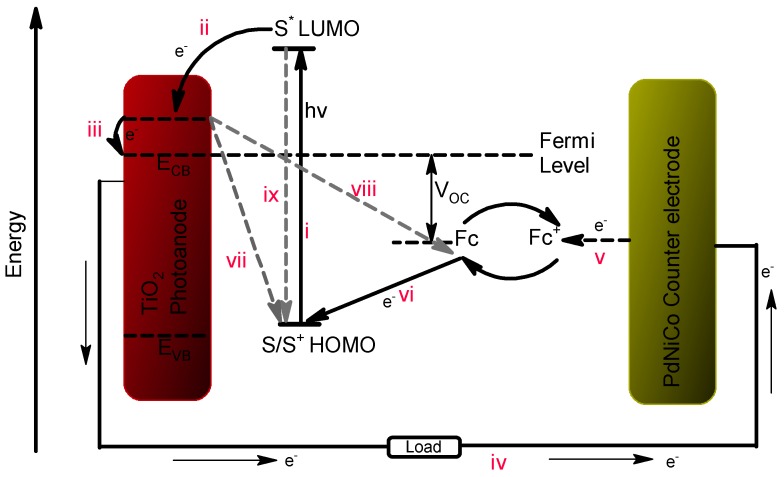
Operational procedure of a dye sensitized solar cell.

The dye sensitized solar cell (DSSC) has been known as one of the most promising thin film solar technologies since it offers considerable advantages compared to the other thin film technologies, most notably its low fabrication cost, and minimal environmental degradation [1]. Despite all the outlined advantages the dye sensitized solar cell is considerably hampered by the presence of ruthenium in the dye sensitizer, and platinum in the counter electrode as well as its low power conversion efficiency. These two metals make up almost half of the total cost of fabrication of a DSSC [1]. As a result, focus has been directed towards improving the efficiency of the DSSC through replacing its existing components or improving the function of the existing ones. Several viable pathways include the addition of CdS quantum-dot in TiO_2_ layer [2], manipulating the morphology of the TiO_2_ electrode so it becomes a nanourchin structure [3] implementing other photoanodes [4], replacement of the iodine electrolyte, development of a dye that can absorb light from the whole visible spectrum as well as the infrared region [3,5,6] and development of an inexpensive platinum free counter electrode that surpasses the platinum efficiency [7,8,9]. The counter electrode is one of the vital components of a DSSC, it is composed of a thin catalyst film attached to the fluorine doped tin oxide (FTO) glass substrate. Its main function is to facilitate the catalytic reduction of the electrolyte, which in turn reduces the oxidized dye molecule thus ensuring electron transportation from the counter electrode to the dye. Figure 1 shows the operational procedure of a DSSC.

Under illumination from the sun dye molecules (S) in the highest occupied molecular orbital (HOMO) absorb photons from the sun and their electrons become excited. These excited molecules (S*) subsequently move to the lowest unoccupied molecular orbital (LUMO) of the dye provided the energy gained from the photons is large enough to overcome the energy difference between the HOMO and the LUMO (i). The excited molecules S* then releases photoelectrons (e^−^; ii), which are then transferred to the conduction band (CB) of the titanium dioxide photoanode, leaving dye sensitizer holes (S^+^; iii). The injected electrons (e^−^) are subsequently led to the external circuit where they migrate towards the counter electrode (iv). At the counter electrode the electrons are stripped (v) by the oxidized ferrocenium ion (Fc^+^) in the electrolyte thereby itself being reduced to ferrocene (Fc). The reduced ferrocene ions then transfer the electrons back to the oxidized dye (vi) molecule reducing it in the process whilst becoming oxidized back to the ferrocenium ion. The transfer of electrons from the counter electrode to the dye by the ferrocene ion thus facilitates regeneration of the dye molecule and completion of the cycle of sunlight conversion into electricity. 

Hence counter electrode fabrication materials must be good electrocatalysts for the electrolyte redox couple as well as being good electron conductors. Platinum is the most widely used and preferred catalyst for counter electrodes (CE) in DSSC, because of its high electrocatalytic ability and conductivity. However, the high cost of platinum, and its low resistance to iodine corrosion makes it almost impossible for large scale fabrication of DSSC. Several materials have been investigated as substitutes for platinum in CE; these include carbon based materials [10], metal sulphides [11,12,13], conducting polymers [14,15] and metal alloys [16,17,18]. Metal alloys in particular have shown great promise with ternary metallic alloys producing modest power conversion efficiencies. The high catalytic capability of binary and ternary alloys has been attributed to the synergy created by the existence of two different atoms in a lattice structure, which is believed to result in charge transfer thus enhancing catalytically active sites [19]. Yang et al [20] fabricated a PtNiCo alloy counter electrode, which exhibited a reduction current density of 12.24 mA·cm^−2^ as compared to 11.13 mA·cm^−2^ for platinum. Charge transfer resistance for the ternary PtNiCo electrode was 0.64 Ω to 0.75 Ω. Further research into ternary alloy counter electrodes produced a NiCuPt alloy exhibiting a reduction current density, peak to peak potential difference and charge transfer resistances of 14.42 mA·cm^−2^, 439 mV and 0.446 Ω, respectively [21]. Ternary alloys can be fabricated using a variety of techniques including dry plasma reduction [7], galvanic displacement [21], cyclic voltammetry method [22] and hydrothermal synthesis [16]. Despite utilizing expensive autoclaves as well as the inability to view the growth process once started, hydrothermal synthesis is renowned for producing high quality crystals, which could be vital in increasing the catalytic effectiveness of the counter electrode. Since the structure and morphology of the desired catalyst material play a crucial role in determining the rate of adsorption of the electrolyte intermediate on the catalyst surface. As such it is of paramount importance that the surface structure of the fabricated electrocatalyst enhance greater adsorption of the electrolyte in order to facilitate higher rates of its reduction. In search of a better functioning counter electrode this work seeks to evaluate the structural, morphological and electrochemical characteristics of hydrothermally fabricated ternary palladium alloys PdNiCo and PdNiCo-rGO. The properties of the as-synthesized palladium alloys were compared to those of platinum. Since the function of the counter electrode is to facilitate the transportation of electrons from the outer circuit to the electrolyte thereby reducing it in the process thus the effect of the counter electrode will be estimated and determined from the electrochemical data such as charge transfer resistance, reduction current density and peak to peak potential difference. In order to improve the function of the counter electrode a ferrocene/ferrocenium electrolyte will be utilized as the electrolyte so as to eliminate the volatile and corrosive iodine electrolyte. The use of a ferrocene redox electrolyte together with a palladium based metallic alloy counter electrode sample has never been reported before.

## 2. Materials and Methods

**Figure 2:**
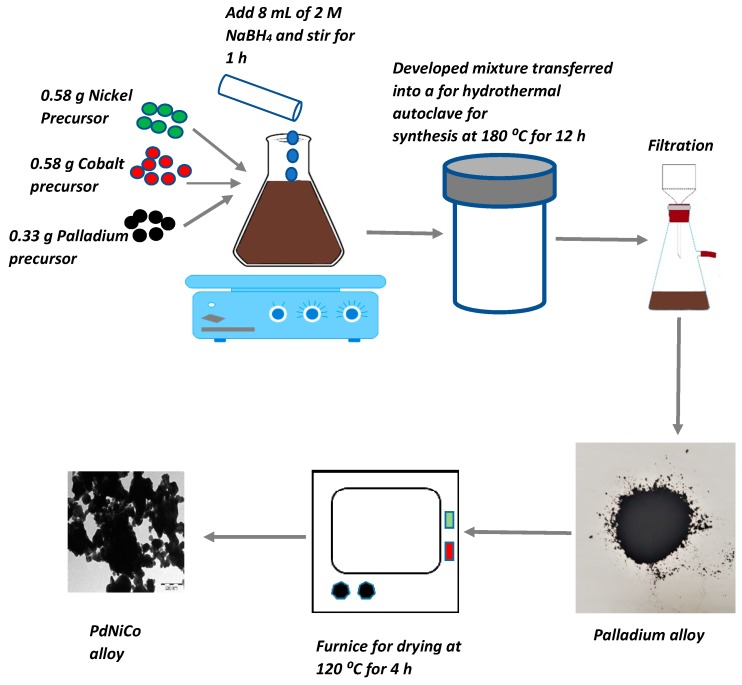
Illustration of the hydrothermal fabrication procedure for the ternary palladium alloy PdNiCo.

All the chemicals utilized in the fabrication of the PdNiCo and PdNiCo-rGO alloys were purchased from Sigma Aldrich (St. Louis, MO, USA) and were used without any further purification. The precursor solutions for the fabrication of the ternary alloys were obtained through dissolving 0.5815 g nickel nitrate hexahydrate, 0.582 g cobalt nitrate hexahydrate and 0.326 g potassium tetrachloropalladate in 50 mL of deionized water. Under vigorous stirring 8 mL of 2 M sodium borohydride was added to the solution resulting in a black mixture. The as-prepared solution was subsequently transferred into a 100 mL autoclave for hydrothermal synthesis at 180 °C for 12 h, which resulted in a black palladium alloy precipitate. The as-synthesized black precipitate was then collected through filtration and washed with deionized water and ethanol so as to remove impurities. The sample was subsequently dried in an oven at 120 °C for 4 h and stored for further analysis. In order to fabricate the carbon supported ternary palladium alloy PdNiCo-rGO 0.5 g of reduced graphene oxide was added to the masses of the other alloy components mentioned above in 50 mL deionized water. Subsequent synthesis stages were similar to those conducted in the fabrication of the unsupported PdNiCo alloy. Figure 2 shows the fabrication procedure for the unsupported ternary palladium alloys.

In order to determine the phase composition of the synthesized ternary alloys, X-ray diffraction (XRD) spectra were obtained using a Bruker D8 Advanced X-Ray Diffractometer (Bruker, Madison, WI, USA). Scanning electron microscopy images for studying the morphological properties of the developed samples were obtained from a field emission scanning electron microscope (FE-SEM) Zeiss Auriga SEM (Carl Zeiss, Oberkochen, Germany) instrument, which was equipped with an eds Smart SEM software. The electrochemical properties of the samples were investigated using a Biologic VMP-300 potentiostat (Knoxville, TN, USA) controlled by the EC-lab V10.37 software. The developed ternary alloy, carbon black and the Ag/AgCl electrodes served as the working, counter and reference electrodes. All electrochemical measurements were conducted in an electrolyte containing 0.06 M ferrocenium hexafluorophosphate, 0.1 M ferrocene and 0.5 M tert-butylpyridine in acetonitrile. Cyclic voltammetry measurements were conducted in the potential range from 0–0.4 V at scan rates ranging from 0–100 mV·s^−1^. Electrochemical impedance spectroscopy (EIS) was conducted in the frequency range from 0.01–100 kHz at 0 V bias with an amplitude of 10 mV.

## 3. Results and Discussion

**Figure 3:**
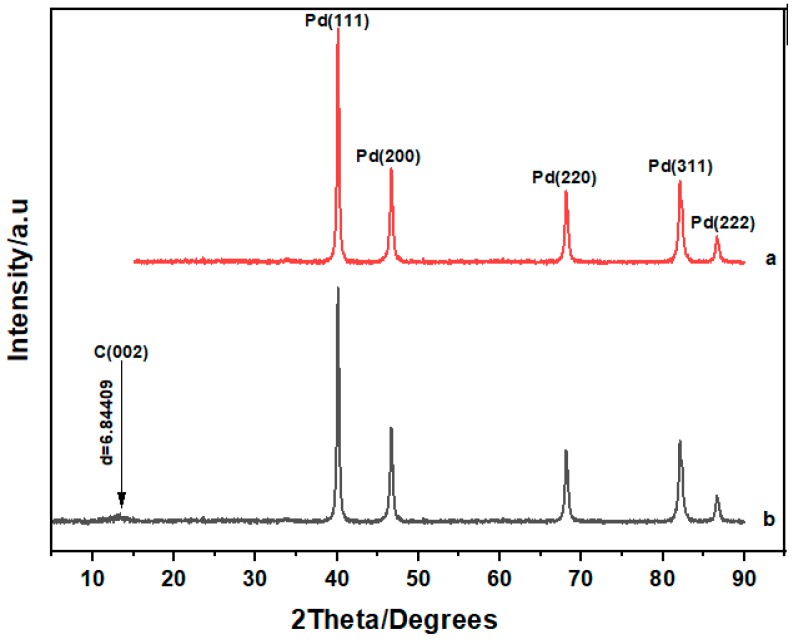
X-ray diffraction patterns for (**a**) PdNiCo and (**b**) PdNiCo-rGO.

Figure 3 depicts the X-ray diffraction (XRD) patterns for the as-synthesized ternary palladium alloys. As shown the diffraction patterns for both alloys were similar with peaks being revealed at 40°, 46°, 68°, 82.5° and 87° corresponding to the (111), (200), (220), (311) and (222) lattice planes of face centered cubic palladium (JCPDS No 46-1043) with space group class (SG) of Fm3m 225. For both samples the peak at 40° exhibited the highest peak signifying that alloy growth was oriented in the (111) plane of palladium. The carbon support in the PdNiCo-rGO sample produced a peak at 14.4°, which was consistent with the (002) plane of carbon. The absence of peaks for both nickel and cobalt signify the successful synthesis of the ternary palladium alloys.

**Figure 4:**
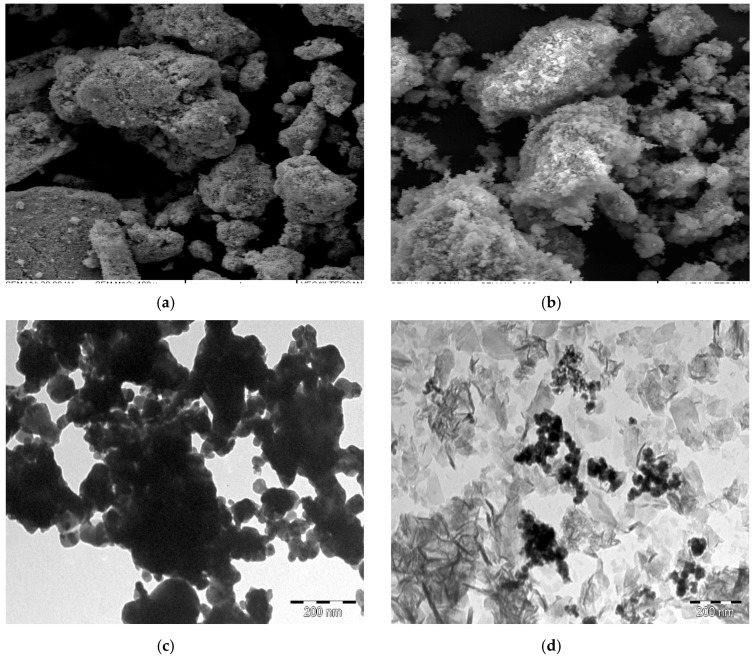
Scanning electron microscopy images of (**a**) PdNiCo and (**b**) PdNiCo-rGO; and transmission electron microscopy images of (**c**) PdNiCo and (**d**) PdNiCo-rGO.

The morphological characteristics of the as-synthesized palladium ternary alloys were investigated using scanning electron microscopy (SEM) analysis. Figure 4a,b show the SEM images obtained at a magnification of 50 micrometers for both PdNiCo and PdNiCo-rGO respectively. Both samples consisted of irregular spherical aggregates. Severe agglomeration was observed for PdNiCo in Figure 4a as compared to PdNiCo-rGO, which exhibited a somewhat limited wider particle distribution relative to that of PdNiCo. The close packed nature of the ternary palladium alloy particles for both PdNiCo and PdNiCo-rGO could potentially limit the access to a wider area for electrolyte infiltration. The limited area of contact between the electrolyte and counter electrode surface limits adsorption thus affecting the rate of reduction of the electrolyte. A limited reduction capacity of the electrolyte slows the regeneration of the oxidized dye molecule thus increasing the probability of electron hole recombination’s with the dye as well as the oxidized redox ion. Further, analysis into the morphological characteristics of the ternary palladium alloys was conducted using transmission electron microscopy (TEM) analysis. As displayed in Figure 4c,d the as-synthesized palladium ternary alloys PdNiCo and PdNiCo-rGO were composed of well defined spherical nanoparticles. Furthermore PdNiCo-rGO in Figure 3b depicts a sea of PdNiCo nanoparticles surrounded by the reduced graphene oxide nanosheets. Average particle size for both alloys was estimated in the range between 9–23.22 nm. Elemental analysis of the as-prepared palladium alloys was conducted using energy dispersive X-ray spectroscopy (EDX). Figure 4e,f show the EDX images for both PdNiCo and PdNiCo-rGO respectively. Both samples were comprised of the constituent elements palladium, nickel, cobalt and carbon.

**Figure 5:**
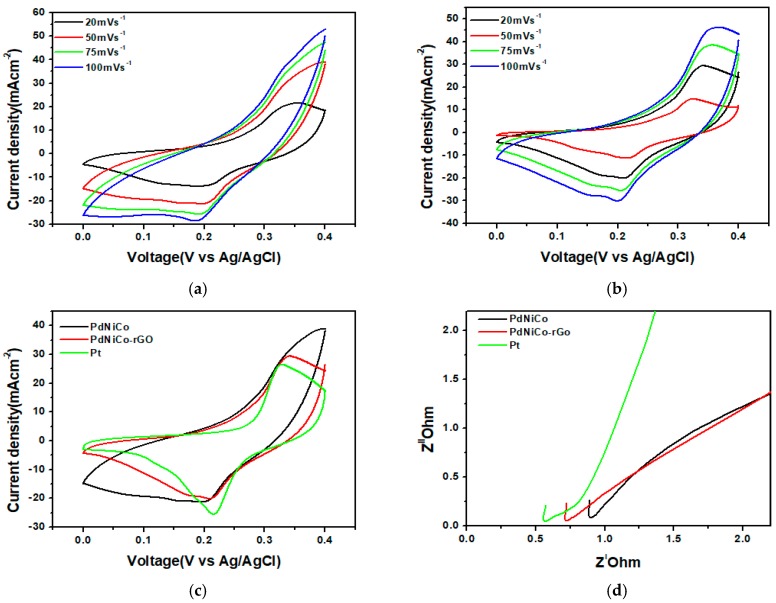
Cyclic voltammetry (CV) analysis curves for (**a**) PdNiCo, (**b**) PdNiCo-rGO, (**c**) comparison of CVs for PdNiCo, PdNiCo-rGO and Pt (platinum) obtained at a scan rate of 50 mV·s^−1^ and (**d**) Nyquist plots recorded at 0 V potential for PdNiCo, PdNiCo-rGO and Pt.

Figure 5a,b depicts the cyclic voltammetry curves for the two palladium alloys PdNiCo and PdNiCo-rGO respectively. Cyclic voltammetry analysis was conducted in a scan rate range from 20–100 mV·s^−1^ profiles for the two palladium alloys at different scan rates. Two significant metrics from cyclic voltammetry (CV) analysis, which were utilized to determine the catalytic effect of the counter electrode sample on the electrolyte was the reduction current density (J_p_) and the peak to peak potential difference (∆E_PP_). The higher the reduction current density the greater is the catalytic capability of the counter electrode catalyst. Whereas the peak to peak potential difference is inversely proportional to the rate of the reduction rate. As a result, a lower ∆E_PP_ signifies a higher rate of reduction of the electrolyte thereby facilitating a faster regeneration of the dye molecule and minimizing electron hole recombinations. Figure 5c shows a comparison of the CV curves of PdNiCo, PdNiCo-rGO and Pt obtained at 50 mV·s^−1^. The reduction current densities for the three samples decreased in the order Pt > PdNiCo > PdNiCo-rGO. Current densities for PdNiCo-rGO and PdNiCo were almost similar at 21 and 23 mA·cm^−2^ respectively despite the addition of reduced graphene oxide, which was intended to shore up catalytic activity through facilitating a greater surface area of contact for maximum interaction between the electrolyte and the counter electrode sample. The observed agglomeration could have potentially minimized the electrolyte adsorption thereby diminishing their catalytic effectiveness. Peak to peak potential difference for the three counter electrode samples was observed at 0.11, 0.12 and 0.19 mV for Pt, PtNiCo-rGO and PtNiCo respectively. A higher catalytic reduction process was thus observed when the platinum sample was used as the counter electrode. The relative similarity between the values for Pt and PdNiCo-rGO gives an indication that the intensity of their catalytic reduction processes was very similar. Comparison of how the counter electrode samples impact electron transfer between the counter electrode surface and the electrolyte was established from the electrochemical impedance spectroscopy analysis results. Figure 5d shows the EIS Nyquist curves for the three samples. The significant parameter from EIS, which gives a measure of the electron transportation system between the counter electrode and the electrolyte is the charge transfer resistance R_CT_. The greater the resistance, the higher is the impedance to electron flow resulting in higher electron hole recombinations, which leads to low cell efficiency. As a result, the resistance R_CT_ for the counter electrode sample should be minimal so as to facilitate effective electron movement. The semi-circle in the high frequency region of the Nyquist plot determines the charge transfer resistance of the developed samples.

From Figure 5d R_CT_ is minimal for platinum at 0.572 Ω whilst PdNiCo-rGO exhibited 0.726 Ω as compared to 0.9 Ω for PdNiCo. The lower charge transfer resistance for PdNiCo-rGO as compared to PdNiCo was attributed to the reduced graphene oxide, which possesses excellent carrier mobilities thus facilitated a higher rate of electron transfer as compared to PdNiCo. Nevertheless, the resistances for both palladium samples were higher than for platinum indicative of its better electrical conductivity. The results obtained vary from those reached by Yang et al. [20] who experienced an increase in reduction current density from 11.13 mA·cm^−2^ to 12.24 mA·cm^−2^ when the platinum counter electrode was replaced by the PtNiCo alloy counter electrode in an iodine electrolyte. The huge variation in the reduction current densities between the PtNiCo/iodine electrolyte combination developed by Yang et al. [16] to the as prepared PdNiCo/ferrocene electrolyte suggests that alternative and viable alternatives to the corrosive iodine electrolyte could be implemented. Further information regarding the stability of the ferrocene electrolyte over a prolonged period of time would be required to ascertain its ability to be a convenient replacement. The significant drawback, which could have potentially minimized the effect of the palladium alloy counter electrode samples, was the reduction in effective surface area for catalytic activity, which was caused by the severe agglomeration both samples underwent.

**Figure 6:**
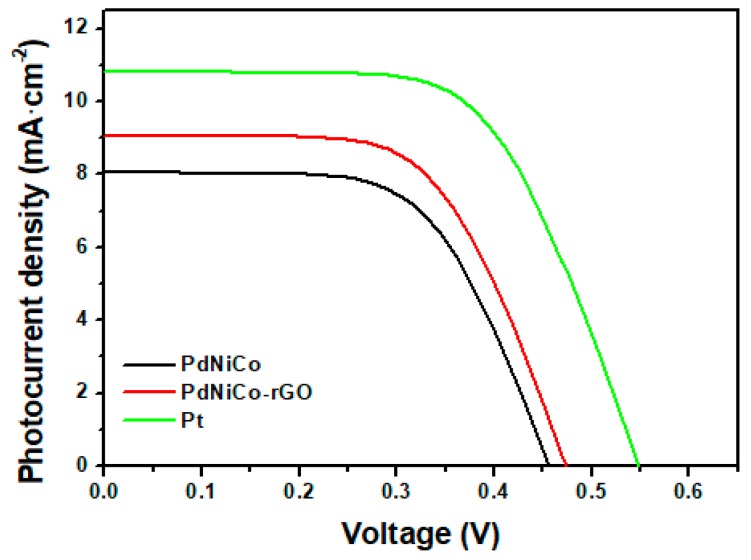
Current-Voltage (J–V) characteristics for the dye sensitized solar cell (DSSC) comprising the ternary palladium alloys and the platinum counter electrode.

Figure 6 shows the J–V characteristics for the developed ternary palladium alloy PdNiCo and its carbon supported form PdNiCo-rGO. Table 1 shows the cumulative photovoltaic results obtained from the analysis conducted. The dye sensitized solar cell developed with the PdNiCo0rGo counter electrode exhibited a 4.36% power conversion efficiency (PCE; V_oc_ = 0.473 V, J_sc_ = 9.06 mA∙cm^−2^ and fill factor (FF) = 54.9%) as compared to the 3.35% efficiency yielded by the DSSC comprising the unsupported PdNiCo alloy. Under similar conditions, the DSSC fabricated utilizing a platinum counter electrode yielded the highest photon to electron generation efficiency at 5.7% (V_oc_ = 0.539 V, J_sc_ = 10.8 mA∙cm^−2^ and FF = 58.1%). The higher open circuit voltage and short circuit current for PdNiCo-rGO, which is almost equivalent to that of platinum was attributed to the greater catalytic reduction of the ferrocene (Fc^+^) ion to ferrocenium (Fc), thereby facilitating a faster regeneration of the oxidized dye molecules and an increased rate of continuation of the photon to the electron generation process by the dye. Resultantly a greater number of electrons were generated, which were transferred through the titanium dioxide photoanode leading to higher short circuit current. Although open circuit voltage was determined by the difference in potential between the Fermi level of the titanium dioxide photoanode and the electrode potential of the ferrocene electrolyte, the lower open circuit voltage for the PdNiCo could be attributed to greater electron hole recombinations caused by the higher charge transfer resistance at the counter electrode.

## 4. Conclusions

Ternary palladium alloys PdNiCo and PdNiCo-rGO were synthesized via a simple hydrothermal procedure. Investigations into the electrochemical reduction capability of the as-synthesized palladium alloys were conducted in a ferrocene redox electrolyte and compared to a platinum sample. Both palladium based alloys were observed as consisting of heavily agglomerated spherical particles as revealed by the SEM and TEM analysis. The catalytic reduction performance of platinum was highest with a reduction current density of 27 mA·cm^−2^ as compared to 22 and 21 mA·cm^−2^ for PdNiCo and PdNiCo-rGO respectively. Peak to peak potential difference for PdNiCo-rGO was closely matched to that of platinum at 0.12 V as compared to 0.11 V signifying that the rate of the catalytic reduction for both samples was closely similar. Better electron transportation from the electrode surface to the electrolyte was observed for PdNiCo-rGO with 0.726 Ω charge transfer resistance being observed. The high catalytic activity of the PdNiCo-rGO counter electrode yielded high power conversion efficiency, short circuit current and open circuit voltage of 4.36%, 9.06 mA∙cm^−2^ and 0.473 V respectively as compared to 5.7%, 10.8 mA∙cm^−2^ and 0.54 V for the best performing platinum based dye sensitized solar cell. Obtained results show that the carbon supported palladium alloy PdNiCo-rGO could potentially replace platinum as the counter electrode catalyst since its electrochemical and photovoltaic performances mirror the efficiency of the platinum electrode. The obtained reduction current density of 21 mA·cm^−2^ for PdNiCo-rGO with a ferrocene electrolyte is significantly higher than that reported for the iodine electrolyte with a PtNiCo electrolyte at 12.24 mA·cm^−2^ [16] signifying that ferrocene could be a viable alternative to the volatile iodine electrolyte. The high degree of agglomeration for both alloys potentially reduced their surface area for interaction with the electrolyte thus limiting the availability of active sites for catalytic activity.

## Figures and Tables

**Table 1:** Electrochemical and photovoltaic parameters for the fabricated DSSCs.

CE	J_red_ (mA∙cm^−2^)	E_PP_ (V)	R_CT_ (Ω)	V_oc_ (V)	J_SC_ (mA∙cm^−2^)	FF (%)	PCE (%)
PdNiCo	22	0.19	0.9	0.449	8.01	54.9	3.35
PdNiCo-rGO	21	0.12	0.726	0.473	9.06	56.3	4.36
Pt	27	0.11	0.572	0.539	10.8	58.1	5.7
